# Breast cancer radiotherapy and the risk of lung injury: Advances and perspectives

**DOI:** 10.36922/arnm025340041

**Published:** 2026-01-21

**Authors:** Shubhankar Suman

**Affiliations:** Department of Oncology, Lombardi Comprehensive Cancer Center, Georgetown University Medical Center, Georgetown University, Washington, District of Columbia, United States of America

**Keywords:** Breast cancer radiotherapy, Radiation-induced lung injury, Radiation pneumonitis, Radiation-induced lung fibrosis, Tamoxifen, Senotherapeutics

## Abstract

Breast cancer is the most prevalent malignancy among women worldwide, and radiotherapy (RT) plays a central role in reducing local recurrence and improving survival. Technological advances such as three-dimensional conformal RT (3D-CRT), intensity-modulated RT (IMRT), volumetric-modulated arc therapy (VMAT), and particle therapies have enhanced dose conformity and reduced exposure to surrounding healthy tissues, particularly the lungs. Nevertheless, radiation-induced lung injury (RILI) remains a clinically relevant concern because of the close anatomical relationship between the breast and lung. RILI is a biphasic process, comprising early radiation pneumonitis and late radiation-induced pulmonary fibrosis, with severity influenced by dose distribution and treatment modality. While 3D-CRT carries a moderate risk due to limited beam modulation, IMRT and VMAT improve target coverage but may increase low-dose exposure to larger lung volumes, potentially increasing the risks of subclinical injury and, in the long term, secondary malignancy. Adjunctive lung-sparing strategies, including deep inspiration breath-hold and image-guided techniques, further reduce pulmonary dose. Proton beam therapy, particularly pencil beam scanning, offers additional lung protection through Bragg peak–based dose deposition, minimizing exit dose and irradiation of non-target tissues. Early clinical data suggest a lower incidence of RILI with PBT, although long-term outcomes remain under investigation. Carbon ion RT remains investigational in breast cancer. This review summarizes current evidence on RILI risk across modern RT modalities. A deeper understanding of modality-specific risks is essential for guiding personalized treatment planning and implementing effective lung-sparing strategies.

## 1. Introduction

Breast cancer is the most frequently diagnosed malignancy among women worldwide, with an estimated 2.3 million new cases and over 670,000 deaths in 2022, making it the leading cause of cancer-related morbidity and mortality in women globally.^1,2^ In the United States, the American Cancer Society projects 316,950 new cases of invasive breast cancer and 42,170 related deaths in 2025, positioning it as the second leading cause of cancer mortality among women, after lung cancer.^3,4^ Advances in screening, diagnosis, and treatment have led to steady improvements in breast cancer survival rates across most countries, with 5-year relative survival exceeding 80% in many regions.^5,6^ Approximately 60% of patients with breast cancer are estimated to receive adjuvant radiotherapy (RT) as part of multidisciplinary management for early-stage and locally advanced disease, most commonly following breast-conserving surgery and in selected post-mastectomy cases.^7–9^ Despite substantial advances in RT techniques ranging from three-dimensional conformal RT (3D-CRT) to more conformal modalities such as intensity-modulated RT (IMRT), volumetric modulated arc therapy (VMAT), and particle-based (proton or carbon-ion) beam therapy, radiation-induced lung injury (RILI) remains a clinically significant, dose-limiting toxicity that can adversely impact quality of life, pulmonary function, and long-term survival.^10^ Its occurrence is influenced by multiple factors, including the irradiated lung volume, mean lung dose (MLD), fractionation schedule, concurrent systemic therapies (e.g., taxanes, trastuzumab, and immunotherapy), pre-existing pulmonary disease, smoking history, and individual radiosensitivity.^10,11^

Pathophysiologically, RILI occurs through two interconnected phases: (i) acute phase, commonly termed radiation pneumonitis (RP), which typically manifests within weeks to a few months after RT. RP may present as asymptomatic radiographic changes or as a symptomatic inflammatory response characterized by cough, dyspnea, low-grade fever, and, in severe cases, hypoxemia. Although RP is often self-limiting or responsive to corticosteroid therapy, it can predispose patients to subsequent chronic injury. (ii) Chronic phase, referred to as radiation-induced pulmonary fibrosis (RIPF; also termed radiation-induced lung fibrosis [RILF]), represents an irreversible stage characterized by permanent remodeling of lung architecture.^11^ The process is driven by the activation and proliferation of myofibroblasts, excessive extracellular matrix (ECM) deposition, and distortion of alveolar structures.^12,13^ Persistent low-grade inflammation, aberrant wound-healing responses, and ongoing oxidative stress contribute to the progressive loss of lung compliance and function. Beyond functional impairment, RIPF creates a pro-oncogenic microenvironment marked by chronic inflammation, cytokine dysregulation (e.g., elevated transforming growth factor-beta [TGF-β], interleukin 6 [IL-6]), and persistent DNA damage.^14–19^ This milieu has been implicated in second primary lung cancer development, particularly in long-term survivors. While the absolute risk of lung cancer after breast RT is relatively low, it appears to be dose-dependent and synergistically amplified by coexisting carcinogenic exposures such as tobacco smoking.^20–22^ The risk is greater in patients who received higher doses to larger lung volumes, those treated at younger ages, and those surviving many years after therapy. Radiation-associated lung malignancies may arise as primary lung cancers induced by mutagenic radiation injury, or present as pulmonary metastases from breast cancer (or less commonly, metastases from another primary). Given the growing number of breast cancer survivors worldwide,^5,6^ it is critical to minimize the incidence of RIPF and subsequent lung malignancy. This review provides a comprehensive synthesis of current evidence on the incidence, pathophysiology, and clinical implications of RILI in breast cancer survivors, with an emphasis on modality-specific risks and emerging technologies aimed at reducing pulmonary toxicity.

## 2. RT modalities in breast cancer treatment and management

RT remains an essential component of multidisciplinary breast cancer management, offering proven reductions in local recurrence and improvements in disease-specific and overall survival across early-stage, locally advanced, and post-mastectomy settings. Over the past three decades, technical innovations have transformed breast RT from two-dimensional approaches to highly conformal, image-guided modalities capable of sculpting dose around complex target volumes while limiting irradiation to surrounding normal tissues, including the lungs.^23,24^ 3D-CRT, historically the standard of care, uses forward-planned tangential fields shaped to the breast or chest wall, with or without regional nodal irradiation (RNI). While this technique achieves acceptable target coverage in many cases, its limited beam modulation capacity can result in suboptimal conformity around irregular anatomy, leading to incidental exposure of adjacent lung parenchyma, particularly when internal mammary nodes are included.^25,26^ To address these limitations, IMRT and its rotational variant, VMAT, have been widely adopted.^27,28^ Both employ inverse planning to modulate beam intensity across multiple angles, enabling superior target conformity, improved dose homogeneity, and better coverage of complex structures such as the chest wall with RNI.^27,29,30^ However, the improved conformity often comes at the cost of a larger “low-dose bath” to surrounding tissues, increasing the volume of ipsilateral and contralateral lung receiving low radiation doses, which may have implications for subclinical RILI and, in long-term survivors, secondary malignancy risk.^27,31,32^ Consequently, the integration of lung-sparing adjuncts, including deep inspiration breath-hold (DIBH), surface-guided RT, and image-guided RT, has become routine in many centers, particularly for left-sided breast cancer, to minimize cardiopulmonary exposure.^33–35^

In addition to ongoing developments in IMRT/VMAT, the advent of particle therapy has introduced new opportunities for further sparing of lung tissue.^36^ Pencil beam scanning (PBS) proton radiation therapy (PRT), an advanced form of proton beam delivery, uses magnetically steered, narrow proton “pencils” to deposit dose spot-by-spot and layer-by-layer, creating highly conformal plans with minimal exit dose beyond the target. This is especially advantageous in scenarios involving internal mammary node irradiation, bilateral disease, or challenging thoracic anatomy, where the mean heart dose and ipsilateral lung V20 (percentage of lung volume receiving ≥20 Gy) can be significantly reduced compared to IMRT/VMAT.^28,37,38^ Clinical series and early-phase trials have reported favorable dosimetric profiles and promising reductions in estimated normal tissue complication probabilities for cardiopulmonary endpoints, though robust, long-term outcome data are still awaited.^28,39–41^ Carbon ion RT (CIRT) represents another advancement in particle therapy, delivering high linear energy transfer radiation with enhanced relative biological effectiveness (RBE) and sharp distal dose fall-off achieved through a spread-out Bragg peak. While CIRT offers theoretical advantages for local control and normal tissue sparing, its role in breast cancer remains largely investigational, with clinical application restricted to a small number of specialized centers in Japan, Germany, and other selected regions.^42–44^

Irrespective of the beam type (photon vs. particle beam) used in RT, the total prescribed dose is conventionally measured in Grays (Gy), which quantifies the physical energy deposited per unit mass of tissue. However, different types of radiation vary not only in physical dose delivery but also in their biological effectiveness, i.e., their capacity to induce cellular damage that mediates both tumor control and normal tissue injury.^45^ This includes stochastic effects such as radiation-induced carcinogenesis, which can occur even at low doses, making biological dose considerations particularly relevant when evaluating long-term pulmonary and secondary cancer risks.^20,23,26,31^ To account for RT modality-dependent biological impact, the concept of RBE is used. RBE is defined as the ratio of the dose of a reference radiation (typically photons) to the dose of the test radiation that produces the same biological effect. For conventional photon-based techniques such as IMRT, the RBE is standardized at 1.^46,47^ PRT, which delivers charged particles with higher linear energy transfer than photons and induces denser ionization and more complex DNA damage, has a clinically adopted RBE of approximately 1.1.^47^ Accordingly, proton doses are expressed as Gy(RBE), calculated by multiplying the physical dose by RBE, allowing meaningful comparison with photon doses.^9^ CIRT, which exhibits substantially higher linear energy transfer than protons, is associated with a correspondingly higher RBE, typically ranging from 2 to 3 depending on tissue type and biological endpoint.^28,43^ This adjustment ensures that the biological impact of PRT and CIRT is accurately represented and compared to photon doses in IMRT/VMAT (Table 1).

Beyond external-beam advancements, several technique-specific approaches also contribute to reducing pulmonary exposure. Accelerated partial-breast irradiation (APBI), delivered through either external-beam modalities or brachytherapy (e.g., interstitial or balloon-based techniques), limits the high-dose region to the lumpectomy cavity with steep dose fall-off, markedly reducing incidental lung dose compared with whole-breast or RNI.^7^ Although APBI is applicable only to selected early-stage patients, its dosimetric profile supports a substantially lower risk of RILI relative to conventional whole-breast irradiation. Collectively, these modality-specific differences in dose distribution, conformity, temporal delivery characteristics, and normal tissue sparing underpin variations in the risk and presentation of RILI among breast cancer patients. Understanding the physical principles and clinical trade-offs of each technique is therefore critical in tailoring RT to individual patients, balancing local control with the minimization of pulmonary toxicity.

## 3. Pathophysiological hallmarks of RILI

RILI encompasses a spectrum of pathological changes ranging from acute inflammation to chronic fibrosis, and in rare cases, may contribute to an increased risk of neoplastic transformation.^48,49^ These pathological states are marked by distinct histopathological and molecular signatures, reflecting complex interactions between irradiated lung parenchyma, resident cells, and the immune system.^50^

Radiation pneumonitis represents the early inflammatory response of the lung parenchyma to exposure to ionizing radiation (IR), typically occurring within 1–6 months after treatment. In breast cancer, the reported incidence of clinically significant RP generally ranges from ~1% to 13%, although higher or lower values have been described depending on study design, diagnostic criteria, era of treatment, and dosimetric characteristics.^51,52^ Histopathologically, RP is characterized by acute injury and apoptosis of type I pneumocytes, which are essential for efficient gas exchange. In response, type II pneumocytes, responsible for surfactant production and epithelial repair, undergo compensatory hyperplasia and may display cellular atypia. Microvascular injury plays a pivotal role in RP pathogenesis. Increased endothelial permeability leads to interstitial and intra-alveolar edema, with fluid accumulation in alveolar septa and airspaces. This is accompanied by a prominent inflammatory infiltrate composed predominantly of mononuclear cells (lymphocytes, monocytes, and macrophages), with neutrophils in early stages, resulting in alveolitis.^53^ Severe cases show hyaline membrane formation, composed of eosinophilic proteinaceous material along alveolar ducts and spaces, indicating extensive epithelial barrier disruption. Vascular congestion, perivascular inflammatory cuffs, and endothelial apoptosis are frequently observed. At the molecular level, RP is orchestrated by an inflammatory cytokine cascade involving tumor necrosis factor-alpha (TNF-α), IL-1β, IL-6, and chemokines such as C–C motif ligand 2 (CCL2) and C–X–C motif ligand 8 (CXCL8), which mediate leukocyte recruitment and activation.^54^ Activation of nuclear factor-kappa B (NF-κB) and oxidative stress pathways further amplifies tissue injury.

**Figure 1.**
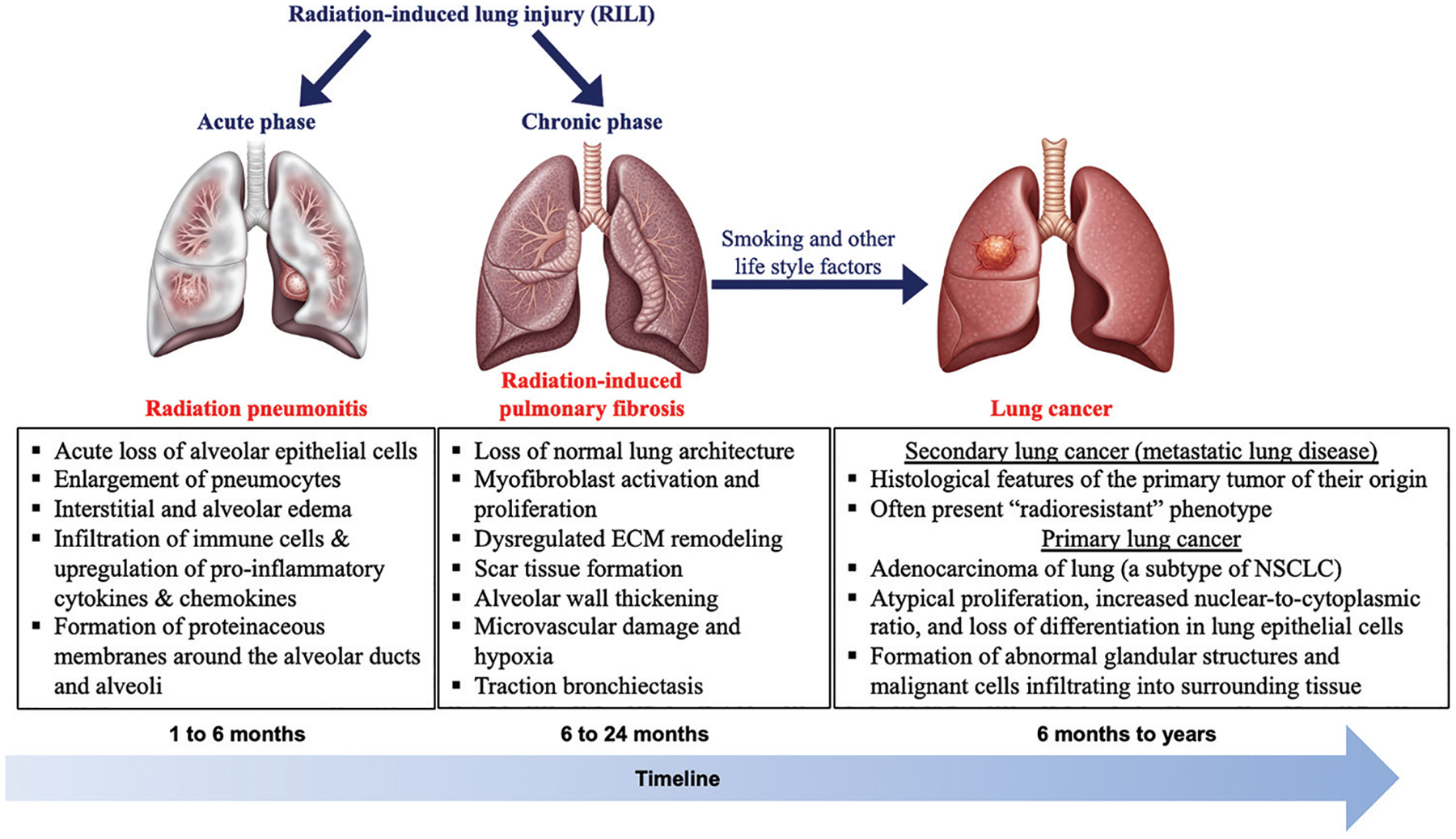
Illustration of pathological progression and associated histopathological hallmarks of radiation-induced lung injury and its potential progression to lung cancer. Initial lung image with pneumonitis, fibrosis, and lung cancers was created using Gemini (Google LLC) and subsequently edited, labeled, and annotated by the author using Adobe Illustrator and Microsoft PowerPoint. Abbreviations: ECM: Extracellular matrix; NSCLC: Non-small cell lung cancer.

Radiation-induced pulmonary fibrosis represents the late, often progressive phase of RILI, typically manifesting 6–24-month post-irradiation. Its incidence in breast cancer varies from 1% to 40% of patients, largely depending on the type of study (retrospective vs. prospective), diagnostic criteria, follow-up duration, and radiation technique.^10,55,56^ RIPF is driven by an aberrant wound-healing response, in which normal parenchyma is replaced by dense ECM rich in type I and III collagen, fibronectin, and proteoglycans. Specifically, RIPF is driven largely by epithelial-mesenchymal transition (EMT) triggered by IR. At the molecular level, IR generates reactive oxygen species (ROS), which activate the ROS/extracellular signal-regulated kinase pathway and promote Snail-G9a complex formation, leading to epigenetic suppression of E-cadherin via H3K9 methylation.^57^ Heat shock protein 27, forkhead box protein M1, and various microRNAs further regulate EMT: suppression of miR-541–5p, miR-486–3p, and miR-155–5p, along with the activation of sphingosine 1-phosphate receptor 3/TGF-β1 signaling, enhances Snail/Slug expression and fibrosis progression.^58^ Excess ROS also activates the NACHT, LRR, and PYD domains-containing protein 3 (NLRP3) inflammasome, increasing IL-1β and driving fibroblast-to-myofibroblast differentiation. Finally, radiation-induced lactate secretion by fibroblasts acidifies the extracellular milieu, activating TGF-β and perpetuating fibrosis. Immune modulation and the persistent activation and proliferation of fibroblasts and their differentiation into myofibroblasts play a central role in contributing to ECM overproduction and tissue contraction during fibrosis progression,^59,60^ where regulatory T cells and M2-polarized macrophages produce pro-fibrotic cytokines (e.g., IL-4 and IL-13) and TGF-β, amplifying EMT and ECM deposition.^61^ Additionally, cellular senescence in macrophages and type II alveolar cells promotes RIPF through the senescence-associated secretory phenotype (SASP), which releases TNF-α, IL-1a, and IL-13, further stimulating M2 macrophages.^62,63^ TGF-β1 signaling is considered the master regulator of radiation-induced fibrogenesis, sustaining myofibroblast activation and *ECM* gene transcription long after the initial injury.^64,65^ Additional contributors include connective tissue growth factor, platelet-derived growth factor (PDGF), and endothelin-1.^58,66^ Architecturally, fibrosis leads to the loss of lung volume, mediastinal shift toward the irradiated side, hemidiaphragm elevation, and displacement of hilar structures. Traction bronchiectasis arises from fibrotic retraction of lung parenchyma. Thickening of alveolar walls and obliteration of capillary beds result in impaired gas exchange and chronic hypoxia, perpetuating the fibrotic cycle (Figure 1). Most cases of RIPF following breast RT are subclinical or mild, detected only on follow-up imaging as focal scarring and volume loss within the irradiated field; however, in severe cases, restrictive lung disease and hypoxemia can occur.

## 4. RILI following breast cancer RT

Breast RT inevitably delivers some degree of incidental dose to the ipsilateral lung; however, modern RT techniques consistently report a low incidence of symptomatic RP, typically in the range of 1–5%. Residual variability across studies largely reflects differences in diagnostic criteria, patient selection, and the transition from older, less conformal approaches to contemporary planning and delivery techniques.^10,67^ Asymptomatic radiological changes are more frequently observed than clinically significant symptoms. Irradiation of regional lymphatic nodes, particularly the internal mammary and supraclavicular nodes, has been linked to greater lung dose exposure and, consequently, an elevated risk of RILI.^10,68,69^ A meta-analysis of breast cancer patients treated with 3D-CRT reported low-grade RP in 22–62% of cases, with a median frequency of ~42%.^70^ Although IMRT may increase the volume of lung receiving low-dose radiation (potentially contributing to subclinical RILI),^71,72^ the incidence of clinically significant RP is lower than with older techniques.^73^ Most RP events are Grades 0–1, presenting as asymptomatic or mild symptoms without functional impairment. Grade 2 RP, requiring corticosteroid intervention, is infrequent, and severe RP (Grades 3–4) is exceptionally rare in breast cancer patients treated with IMRT. For example, in a prospective study (2010–2013) using multibeam IMRT in 113 patients, the median lung V5 was 100% and V20 was 29%.^74^ Respiratory toxicity occurred in only 10.6% of patients (11/104), with a single Grade 3 RP event (0.96%). No significant changes were seen in pulmonary function tests or pneumonitis scores post-treatment. However, the median follow-up (~4.5 years) may not capture late-onset RILI.

Meanwhile, PRT, particularly with PBS techniques, is an emerging modality for early-stage and locally advanced breast cancer. Dosimetric and early clinical studies indicate that PRT can reduce both early and late lung toxicities.^75,76^ Comparative planning studies consistently demonstrate superior target coverage and reduced doses to organs-at-risk compared with photon-based IMRT or VMAT.^77,78^ Evidence from other thoracic malignancies suggests potential benefits. For example, randomized trials in non-small cell lung cancer did not show significant reductions in Grade ≥3 RP or local failure with protons versus IMRT, but did demonstrate improved cardiac sparing.^79^ The particle therapy cooperative group consensus statement notes that, despite strong dosimetric advantages, current evidence does not yet provide Level 1 or 2 data demonstrating superior clinical outcomes for PRT over IMRT in breast cancer, including statistically significant reductions in symptomatic RP.^80^ The consensus recognizes PRT’s potential for enhanced normal tissue sparing, mainly in high-risk, anatomically complex scenarios (e.g., left-sided chest wall with nodal irradiation, bilateral disease, and re-irradiation). PRT is still evolving and moving toward robust, model-based, and trial-driven validation of PBT strategies, encouraging enrollment in trials, and harmonizing technical standards across centers to improve evidence quality. Ongoing randomized trials and extended follow-up are expected to clarify the long-term incidence of RILI and help identify breast cancer subgroups that may derive the greatest clinical benefit from PRT.^80^

## 5. Dosimetric predictors and clinical risk factors for RILI in breast cancer RT

Dosimetric parameters are pivotal in both predicting and mitigating the risk of RILI, particularly in modern breast cancer RT, where precision targeting and sparing of organs-at-risk are paramount. The most validated predictors of pulmonary toxicity are the MLD and the volume of lung receiving a given threshold dose (Vx Gy), with V20 being the most clinically relevant. An MLD <15 Gy and V20 < 30% are consistently associated with a lower likelihood of RILI, whereas V20 > 30% significantly increases the risk of clinically relevant pneumonitis and late fibrosis.^81,82^ MLD values exceeding 20 Gy have been correlated with an approximately 20% incidence of symptomatic pneumonitis.^81^ Notably, V5 > 50% increases the “low-dose bath” to the lungs after photon RT and has been linked to higher rates of radiographic lung changes and potential subclinical injury.^81,83^ Conversely, DIBH, particularly in left-sided breast cancer, substantially reduces both lung and cardiac doses, thereby lowering RILI risk.^34,84^ PRT, leveraging the Bragg peak to eliminate exit dose, has been shown to achieve the lowest integral lung dose among all modalities.^85^ A meta-analysis by Gokula *et al*.^70^ supports maintaining V20 < 30% to minimize pulmonary complications. Similarly, limiting MLD to <15 Gy is associated with a reduced risk of both RP and RIPF.^11,86^

Several clinical and treatment-related factors can exacerbate RILI risk. Hypofractionation (larger fraction sizes over fewer sessions) may increase toxicity compared with conventional fractionation.^11,87^ RNI, especially when targeting the internal mammary or supraclavicular nodes, substantially elevates ipsilateral lung V20 and MLD, thus heightening RILI risk.^32,88^ Patient-specific factors also play a crucial role. These include pre-existing pulmonary disease (e.g., chronic obstructive pulmonary disease and interstitial lung disease), advanced age (particularly >50 years), smoking history, higher tumor and nodal stage, and concurrent or recent chemotherapy.^89^ Sequential chemoradiation after breast-conserving surgery usually results in only mild, transient pneumonitis (Grade ≤2). However, tamoxifen combined with the luteinizing hormone-releasing hormone (LHRH) analog goserelin has been associated with a notably increased risk of pneumonitis in breast cancer patients receiving chemoradiation.^90^ Additional contributors, such as thoracic cage anatomical abnormalities, may predispose patients to higher lung doses during treatment (Table 2). Taken together, RILI risk is determined by an interplay between dosimetric thresholds, treatment technique, and individual patient factors. Optimal lung-sparing strategies require an integrated approach that accounts for both quantitative dose metrics and qualitative clinical considerations.

## 6. Subclinical RILI and cancer risk following breast cancer RT

Breast cancer RT can create a pro-inflammatory microenvironment in the lung, which theoretically could influence the initiation or promotion of secondary lung malignancies in breast cancer survivors. It is important to distinguish between second primary lung cancers (arising from native lung epithelial cells) and pulmonary metastases from breast cancer or other primaries. Pulmonary metastases retain the histologic and immunophenotypic features of the primary breast tumor; they are distinct from second primary lung cancers arising from native lung epithelium. The risk of second primary lung cancer after breast RT is generally low. However, it increases with time since treatment, higher irradiated lung volumes, and smoking history. A large Swedish population-based cohort study reported that at 20 years, the cumulative incidence of lung cancer was 3.0% in women treated with RT compared with 2.3% in those who did not receive RT, with excess risk becoming apparent after 5 years and increasing with longer follow-up.^49^ Multiple studies have demonstrated a strong synergistic effect between RT and smoking, with absolute risks several-fold higher in smokers compared to never-smokers.^91–93^ Subclinical RILI, detectable only on imaging or histopathology, may contribute to long-term carcinogenic risk by inducing persistent inflammation, oxidative stress, and genomic instability in lung tissue.^67,89^ Radiation can cause DNA double-strand breaks, chromosomal rearrangements, and mutations in oncogenes (e.g., *EGFR* and *KRAS*) and tumor suppressors (e.g., *TP53*), all of which can promote malignant transformation.^89,94,95^ Absolute long-term risks of modern breast cancer RT in the context of lung cancer incidence were approximately 4% for long-term continuing smokers versus 0.3% for nonsmokers.^89^ IMRT and VMAT often increase the “low-dose bath” to the lung, which in modeling studies has been associated with higher excess absolute risk of lung malignancies compared with 3D-CRT.^96^ However, large registry analyses have not consistently shown higher second cancer diagnoses with IMRT versus 3D-CRT over median follow-ups of 5–10 years.^97^ PRT, particularly PBS, offers distinct dosimetric advantages by eliminating exit dose and reducing integral lung exposure. Cartechini *et al*.^98^ demonstrated that PBS plans for breast cancer with RNI significantly reduced MLD and estimated absolute risk for secondary lung cancer compared with both VMAT and 3D-CRT. Paganetti *et al*.^99^ modeled lifetime attributable risk using patient-specific plans and found that contralateral lung and breast second cancer risks could be markedly reduced with PBS compared to photon-based RT. These benefits are particularly relevant for younger patients with a long life expectancy, in whom even small absolute reductions in risk translate to greater long-term benefit. Overall, while the absolute incidence of second primary lung cancer after breast RT remains low, risk mitigation through lung dose reduction, smoking cessation, and the use of advanced modalities such as DIBH and PRT remains an important component of survivorship care.

## 7. Tamoxifen and RILI risk in breast cancer RT survivors

Tamoxifen, a selective estrogen receptor modulator, remains a key component of adjuvant endocrine therapy for hormone receptor-positive breast cancer, significantly lowering recurrence and mortality.^100,101^ However, its concurrent use with RT has been associated with a heightened risk of RILI, particularly pulmonary fibrosis.^102–104^ Clinical and translational studies suggest that tamoxifen may exacerbate fibrotic changes through the upregulation of TGF-β, a cytokine pivotal in promoting ECM deposition and fibrogenesis.^102,105,106^ Several prospective and retrospective analyses have reported higher rates of clinically significant lung fibrosis when tamoxifen was administered during RT compared to RT alone.^10,107,108^ While the timing of tamoxifen administration (sequential vs. concurrent) has been investigated, most evidence indicates that its impact is mediated via tissue remodeling pathways rather than acute injury, and temporal separation may not fully mitigate risk.

Nonetheless, several pragmatic clinical strategies have been proposed to reduce potential tamoxifen-associated pulmonary toxicity in patients undergoing RT, particularly those with elevated baseline lung dose metrics or pre-existing pulmonary compromise. These include (i) deferring tamoxifen until the completion of RT, while initiating temporary ovarian suppression (e.g., LHRH agonist) in premenopausal women to maintain systemic endocrine blockade during RT; (ii) starting adjuvant endocrine therapy with ovarian suppression ± an aromatase inhibitor during RT, followed by introducing tamoxifen after RT if clinically indicated; (iii) limiting the duration of tamoxifen (e.g., to the initial 2 years) before transitioning to an aromatase inhibitor in appropriate candidates; and (iv) using aromatase inhibitor upfront in postmenopausal women when appropriate, thereby avoiding potential tamoxifen-related enhancement of fibrotic pathways during RT.

These individualized approaches may be particularly relevant for patients at higher risk of RILI due to factors such as large RT volumes, RNI, and compromised baseline lung function. Breast cancer survivors treated with adjuvant RT have a modest but measurable long-term risk of developing a second primary lung cancer, particularly in the ipsilateral lung.^109^ Importantly, population-based analyses have not demonstrated a significant increase in second primary lung cancer incidence attributable to tamoxifen. In fact, some reports suggest that patients who develop second primary lung cancer after receiving anti-estrogen therapy (predominantly tamoxifen) may experience improved lung cancer-specific survival, possibly reflecting systemic antitumor effects.^110^ Overall, current evidence does not support tamoxifen as an independent risk factor for second primary lung cancer. However, its interaction with RT-related pulmonary effects remains an area of ongoing investigation.

Beyond endocrine therapy, the expanding use of targeted and immune-based systemic agents in breast cancer raises new considerations regarding their potential interaction with RT and the risk of RILI. Anti-human epidermal growth factor receptor 2 therapies such as trastuzumab are generally safe in combination with breast RT.^111^ Immune checkpoint inhibitors, increasingly incorporated into early-stage and metastatic triple-negative breast cancer, introduce an additional layer of complexity given their capacity to induce immune-mediated pneumonitis, which may overlap temporally and mechanistically with RILI.^112^ As systemic therapy options continue to broaden, understanding and anticipating how these agents interact with thoracic irradiation will be increasingly important to personalize treatment sequencing, reduce pulmonary toxicity, and maintain optimal oncologic outcomes.

## 8. RILI assessment and emerging therapeutics

Early and accurate assessment of RILI remains a clinical challenge, as its manifestations often overlap with treatment-related pneumonitis, infectious etiologies, and cancer-related pulmonary complications. Diagnostic evaluation relies on a combination of morphologic, functional, and increasingly computational approaches.^113,114^ Pulmonary function tests may demonstrate early restrictive changes, including reductions in lung volumes and diffusing capacity, which frequently precede overt radiographic findings.^114^ While conventional chest radiographs may reveal volume loss or linear scarring within irradiated fields, high-resolution computed tomography (CT) (HRCT) is the gold standard for characterizing RILI across its inflammatory and fibrotic phases. Typical features include ground-glass opacities, patchy consolidation with air bronchograms, volume loss, traction bronchiectasis, and architectural distortion conforming to radiation dose distributions. Positron emission tomography-CT can support differential diagnosis by distinguishing metabolically quiescent radiation fibrosis from fluorodeoxyglucose-avid recurrent or second primary malignancy. Increasingly, imaging acquired during RT, particularly cone-beam CT (CBCT) used for image guidance, offers an opportunity to monitor early subclinical density changes that may signal evolving injury.

Beyond conventional imaging, advanced functional and computational tools are enhancing early detection and risk stratification. Functional imaging modalities, such as single photon emission CT/CT perfusion, dual-energy CT, and hyperpolarized gas magnetic resonance imaging, allow regional mapping of ventilation and perfusion, enabling the identification of high-value lung subunits that may be preferentially spared during treatment.^113,114^ Parallel advances in radiomics and artificial intelligence (AI) now permit the extraction of high-dimensional quantitative features from baseline staging CT, serial HRCT, or even daily CBCT datasets. These radiomic and machine-learning models capture subtle texture, heterogeneity, and voxel-wise dose-response patterns that are not discernible to the human eye and have shown promise in predicting both acute pneumonitis and late fibrosis earlier and with greater accuracy than traditional dose–volume metrics. The integration of longitudinal “delta-radiomics,” dose-coupled feature analysis, and functional-avoidance planning provides a foundation for future adaptive strategies in which treatment plans may be dynamically modified based on early imaging signatures of lung sensitivity.^115,116^ Collectively, the convergence of morphologic imaging, functional assessment, and AI-driven predictive analytics is reshaping the clinical approach to RILI. As breast cancer treatment increasingly incorporates systemic agents with intrinsic lung disease risk and more advanced RT modalities, a refined and multimodal assessment framework will be essential for optimizing patient selection, tailoring dose distributions, and ultimately reducing the long-term pulmonary burden among breast cancer survivors.

Mild RP (Grades 1 and 2) is often managed conservatively with observation, cough suppressants, and bronchodilators. Systemic corticosteroids remain the mainstay for moderate to severe RP (Grade ≥2), with prednisone initiated at 0.5–1.0 mg/kg/day for 2–4 weeks, followed by a gradual taper over 6–12 weeks according to symptomatic and radiographic response. Inhaled corticosteroids, such as budesonide or fluticasone, may serve as adjunctive therapy, particularly for steroid-sensitive phenotypes or during tapering. In steroid-refractory cases, immunosuppressants, including mycophenolate mofetil, azathioprine, and cyclophosphamide, have been employed, though clinical trial data are limited. Optimal outcomes require multimodal strategies accounting for patient-specific factors, lung radiosensitivity, tissue turnover, and dosimetric parameters.^11,12,15^

Radiation-induced lung fibrosis is characterized by progressive respiratory impairment. Supportive measures, including long-term oxygen therapy, pulmonary rehabilitation, and bronchodilators, are critical for maintaining exercise tolerance and quality of life. Repurposing anti-fibrotic agents approved for idiopathic pulmonary fibrosis is under investigation. Pirfenidone, a TGF-β and TNF-α antagonist, and nintedanib, a tyrosine kinase inhibitor targeting vascular endothelial growth factor receptor, PDGF receptor, and fibroblast growth factor receptor pathways, have demonstrated anti-fibrotic efficacy in preclinical RILF models.^117^ Radiation-modifying strategies can be classified as radioprotectors, mitigators, or therapeutic agents, each targeting distinct phases of tissue injury. Radioprotectors are administered pre-irradiation, mitigators during or immediately after exposure, and therapeutic agents post-toxicity to slow or reverse damage.^118^ Amifostine, a thiol-based radioprotector, is clinically approved and has been associated with reduced RP incidence in thoracic RT with a favorable safety profile.^119^ Other agents, including porphyrins (AEOL 10113), cerium oxide nanoparticles, and BIO300 (genistein formulation), similarly attenuate oxidative stress, pro-fibrotic signaling, and vascular damage in animal models.^120–122^ Pentoxifylline, a xanthine derivative, mitigates lung toxicity by enhancing microcirculation, reducing TNF-α, and decreasing vascular resistance by regulating cellular senescence.^123^

Additional evidence has also implicated cellular senescence in the pathogenesis of both RILI and secondary lung carcinogenesis.^124^ Senescent cells, which accumulate after IR, secrete a SASP comprising pro-inflammatory, pro-fibrotic, and pro-tumorigenic factors such as TGF-β, IL-6, IL-1β, and matrix metalloproteinases. Persistent SASP signaling promotes chronic inflammation, fibrosis, and genomic instability,^125–129^ creating a microenvironment conducive to cancer development.^130,131^ Senotherapeutics, targeting these cells, offer a novel strategy to mitigate RILI and long-term malignancy risk.^132^ Senotherapeutics fall into two categories: senolytics, which selectively induce apoptosis in senescent cells by targeting survival pathways, and senomorphics, which suppress SASP without eliminating the cells.^128,129,133^ Preclinical models of thoracic irradiation have demonstrated that senolytic cocktails, such as dasatinib plus quercetin, reduce fibrosis and inflammation, preserve lung architecture, and improve survival. Senomorphic agents such as rapamycin, a mammalian target of rapamycin inhibitor, delay fibrosis progression.^128,129,133,134^

From a clinical perspective, these approaches could be therapeutically integrated to intervene in patients exhibiting early radiographic or functional signs of lung injury. Critical considerations for translation include optimal timing relative to RT, dose scheduling, potential interactions with systemic therapies (e.g., endocrine agents and targeted therapies), and long-term safety in a population already exposed to adjuvant or RNI. Early-phase clinical trials leveraging biomarkers of senescence (such as circulating SASP factors and imaging surrogates) could provide patient stratification tools, enabling precision mitigation of RILI. Overall, senotherapeutics represent a promising frontier for reducing both acute and chronic pulmonary toxicity after breast cancer RT, with the potential to improve long-term lung health and survivorship outcomes pending rigorous clinical validation.

## 9. Conclusion

RILI remains a pivotal consideration in the radiotherapeutic management of breast cancer, despite advances in treatment precision. A comprehensive understanding of RILI’s biphasic trajectory from RP to RILF is essential for optimizing individualized treatment planning and mitigating toxicity. Beyond acute manifestations, long-term pulmonary consequences include persistent fibrosis, reduced pulmonary function, and an elevated risk of second primary lung cancer. These consequences are modulated by multiple factors, including latency periods, baseline lung function, prior smoking history, age at exposure, and interactions with systemic therapies such as tamoxifen. While evidence regarding tamoxifen’s effect on second primary lung cancer remains inconclusive,^135^ its concurrent use with RT has been associated with enhanced fibrotic potential via TGF-β–mediated pathways.^102,105,106^ Given the potential for delayed complications, structured long-term follow-up is imperative. Subclinical RILI may be overlooked, which could lead clinicians to underestimate long-term secondary malignancy risk. Clinicians should maintain vigilance for new pulmonary symptoms and employ individualized screening strategies, guided by patient-specific risk factors. Surveillance protocols should integrate imaging modalities, ranging from chest radiography to HRCT scans for early detection, coupled with regular pulmonary function assessments. Risk stratification should consider both dosimetric parameters and clinical variables, including age, baseline lung function, smoking history, and concurrent systemic therapies. Emerging approaches, such as AI and predictive modeling, hold promise for refining risk assessment and identifying patients at a higher risk of RILI and secondary malignancies.^136–138^ Technological advances in RT, including VMAT, have significantly improved dose conformity and reduced high-dose exposure to normal lung tissue. PRT and CIRT offer further potential for sparing healthy tissue and mitigating pulmonary toxicity, with early data suggesting favorable toxicity profiles. Investigational approaches, such as FLASH RT delivering ultra-high dose rates (≥40 Gy/s), are particularly compelling due to preclinical evidence of normal tissue sparing while maintaining tumoricidal efficacy.^139–141^ The integration of these novel modalities with systemic therapies, including endocrine agents, immunotherapies, and targeted treatments, represents a critical frontier for future research.

Prospective, multi-institutional studies are urgently needed to clarify the long-term pulmonary outcomes associated with PRT, CIRT, and FLASH RT. In parallel, translational research into biomarkers of RILI, including circulating TGF-β, cytokine signatures, and senescence-associated markers, could enable early detection and preemptive intervention. Emerging therapeutics targeting fibrosis, oxidative stress, and cellular senescence—including senolytics and senomorphics—offer opportunities to mitigate RILF and reduce secondary malignancy risk. Integration of these interventions with precision RT may redefine the therapeutic index, optimizing tumor control while preserving long-term pulmonary health. In conclusion, continued innovation in breast cancer RT must be paired with rigorous long-term monitoring and patient-centered care strategies. The central challenge is to achieve sustained oncologic control while minimizing pulmonary injury, thereby improving survival, functional outcomes, and quality of life for the growing population of breast cancer survivors. Future research must emphasize not only technological refinement but also mechanistic understanding, biomarker-guided risk stratification, and combinatorial therapeutic strategies to fully realize this goal.

## Figures and Tables

**Table 1.** A comparison of irradiation parameters commonly used in IMRT, PRT, and CIRT

Parameters	IMRT	PRT	CIRT
Total dose	40–60 Gy	40–60 Gy (RBE)^a^	40–70 Gy (RBE)^b^
Fraction dose	2–3 Gy per fraction	1.8–2.5 Gy (RBE) per fraction	3.0–4.0 Gy (RBE) per fraction
Hypofractionation	Widely standardized with robust evidence (40–42.5 Gy in 15 fractions)	Protocols vary (26–40 Gy (RBE) in 5–15 fractions); increasing interest in ultra-hypofractionation	48–64 Gy (RBE) in 12–16 fractions; Ultra-hypofractionated protocols 45–50 Gy (RBE) in 4–8 fractions
Dose conformity	High	Very high	Very high
Lung dose	Lower than 3D-CRT, but increased V5	Minimal	Minimal
Incidence of RP (%)	<5	<1	<1
Radiation fibrosis	Less extensive	Minimal	Minimal
Long-term lung function	Mild changes possible	Under investigation	Under investigation

Notes:

a In PRT, dose is reported in Gy (RBE**) (**Gray relative biological effectiveness [RBE]) to account for the increased biological potency of protons compared to photons (average RBE~1.1).

b In CIRT, dose is reported in Gy (RBE) to account for the increased biological potency of carbon-ions compared to photons (average RBE 2–3).

Abbreviations: CIRT: Carbon ion radiotherapy; CRT: Conformal radiotherapy; IMRT: Intensity-modulated radiotherapy; PRT: Pencil beam scanning proton therapy; RP: Radiation pneumonitis.

**Table 2.** Risk factors for RILI development after breast cancer radiotherapy

Parameters	Associated risk
V5 (percentage of lung volume receiving >5 Gy) ≤65%	Increased chronic lung injury
V20 (percentage of lung volume receiving >20 Gy) >35%	~7% risk of RILI
Mean lung dose >20 Gy	~20% risk of RILI
Age >50 years	Reduced pulmonary reserve, increased RILI risk
Smoking	Increased RILI risk
Pre-existing conditions (COPD or interstitial lung disease)	Predispose patients to RILI
Concurrent therapies (tamoxifen or chemotherapy)	May influence RILI progression
Anatomical deformities	An uneven radiation field increases lung exposure

Abbreviations: COPD: Chronic obstructive pulmonary disease;

RILI: Radiation-induced lung injury.
